# Supplementary material for: Estimating city-level travel patterns using street imagery: A case study of using Google Street View in Britain
Source: PLoS One. 2018 May 2;13(5):e0196521. doi: 10.1371/journal.pone.0196521 (PMC5931639; doi:10.1371/journal.pone.0196521)
Supplement: S1 File — (DOCX) [file pone.0196521.s001.DOCX]

# S1) Further details on methods and results

## Google Street View API

### Accessing images from Google Street View

The process of obtaining an image for a given location and in a given direction is automated through the use of an application programming interface (API). GSV API is in the form of a web URL which when accessed through a browser displays the corresponding image. The URL includes all the parameters needed to select an image (https://developers.google.com/maps/documentation/streetview/intro). There are 5 parameters in the API—(a) ‘location’ or ‘pano’, (b) ‘heading’, (c) ‘fov’, (d) ‘pov’, (e) ‘size’, and, in addition, API ‘key’.

‘location’ indicates geographical coordinates (latitude, longitude) and can be replaced by ‘pano’ which is the unique ID of the panorama—a 22-character long string. ‘heading’ indicates the compass heading of the camera, and can be specified from 0 to 360 degrees. ‘fov’ (field of view) represents zoom level, ‘pitch’ specifies the up or down angle of the camera relative to the Street View vehicle, and ‘size’ specifies the size of the image. ‘key’ is the confidential API key which is linked with the Google account and through which payment is channelised. We used the default value of fov (90 degrees) and that of pitch (0 degrees) and the maximum size of image (640×640 pixels). For testing different features of GSV and for developing the methodology we used Cambridge city as a test bed from our set of sample cities.

### Metadata

To access metadata of the images, we used GSV API metadata request, which uses geographic coordinates of a location as the only input (https://developers.google.com/maps/documentation/streetview/metadata). The metadata reported by the API includes year and month (yyyy-mm format) of the panorama and its unique ID, referred to as ‘pano_id’ in the output (see Fig A). This is the same information as displayed in the web interface of GSV. The date is shown on the bottom right corner of the image displayed on the web. For instance, for the location shown in Fig A the website shows the following: ‘Image capture: October 2016’. The ID is also included in the URL^^[[1]](#footnote-1)^^ and can be identified as a 22-character string between the two sets of characters— ‘1s’ and ‘!2’, or between ‘panoid%3D’ and ‘%26output’(single quotation marks used only for distinction and are not included in the URL). Thus, when the API is used with a geographic location, it gives the latest image captured for that location.


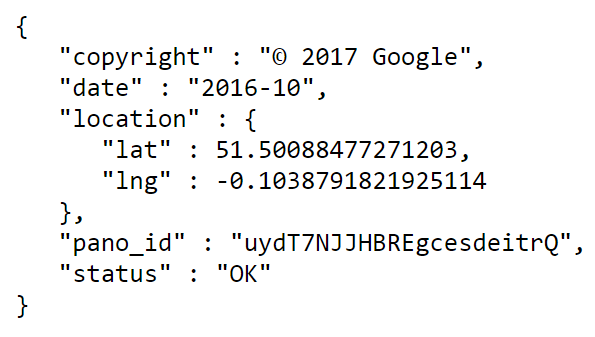


**Fig. A. Output of Google Street View metadata request API**

### Determining time-of-day

Metadata available for GSV images is limited to only month and year. The day of the week as well as time of the day is unknown, both of which are significant variables affecting travel patterns. From general observations of images, it is clear that images are captured during daytime and the time when it is not raining. The two conditions are logical to capture best quality images. While determining day of the week is not possible, we used the angle of shadows to determine an approximate time of day when images were captured. For this, we used the concept of sun-dials. We used an online tool called ‘SunCalc’ (<http://suncalc.net/>) which uses date as an input and has a map on which the location for which time-of-day is to be determined can be selected. By adjusting the angle of sun so that it is parallel to the shadow in the image, an approximate time-of-day can be known.

### Spatial variation in the time stamp of the images

We generated random locations in Cambridge and accessed the metadata corresponding to those. We observed that the year reported in the metadata of the images varied spatially across the city. The outer parts of the city have much higher proportion of older images (2012 or earlier) compared to inner parts, though all locations have a mix of older as well as latest images. We found a similar pattern in many other cities. This indicates that every round of data collection for GSV does not cover the whole city. Thus, using the API with the ‘location’ parameter, the set of images obtained would belong to different years for different locations depending on when the latest images were captured for those locations.

### Accessing Historic images

The ‘streetview’ package uses geographic location of a point as an input. Its output includes the metadata of the most recent as well as historical panoramas available within 5 meters of the location. This output corresponds to what users access through the historic imagery option on the web interface of GSV. For every panorama, the output includes metadata with the same set of information as that obtained by GSV API metadata request (as shown in Fig A). Using this information, panorama IDs of the period of interest can be selected. Next, we use panorama ID in the API (parameter ‘pano’) instead of the ‘location’, thus controlling for the year when images were captured.

### Table A: Yearly distribution of the historic panoramas

(Obtained from 2000 locations in each city and number of panoramas in each location ranges from 0 to 7)

| **PUA** | **2008** | **2009** | **2010** | **2011** | **2012** | **2013** | **2014** | **2015** | **2016** | **2017** |
| --- | --- | --- | --- | --- | --- | --- | --- | --- | --- | --- |
| **Birkenhead** | 403 | 1420 | 0 | 130 | 1229 | 0 | 577 | 663 | 505 | 1 |
| **Birmingham** | 650 | 857 | 348 | 839 | 1324 | 0 | 804 | 556 | 618 | 350 |
| **Blackburn** | 55 | 1596 | 0 | 874 | 522 | 0 | 366 | 492 | 463 | 1 |
| **Blackpool** | 43 | 1474 | 0 | 1 | 398 | 0 | 770 | 404 | 600 | 0 |
| **Bournemouth** | 84 | 1304 | 90 | 950 | 1143 | 2 | 558 | 616 | 685 | 1 |
| **Brighton** | 46 | 1607 | 48 | 961 | 1436 | 1 | 856 | 980 | 880 | 2 |
| **Bristol** | 961 | 537 | 25 | 6 | 1205 | 1 | 737 | 647 | 583 | 323 |
| **Cambridge** | 1157 |  | 709 | 297 | 1437 | 3 | 1609 | 994 | 871 | 1 |
| **Chatham** | 662 | 829 | 45 | 0 | 1445 | 0 | 454 | 623 | 453 | 0 |
| **Crawley** | 188 | 1344 | 0 | 14 | 1595 | 5 | 556 | 771 | 729 | 0 |
| **Hull** | 1223 | 787 | 702 | 31 | 1729 | 0 | 563 | 554 | 516 | 0 |
| **Ipswich** | 1 | 1592 | 224 | 551 | 1660 | 1 | 792 | 825 | 698 | 1 |
| **Leeds** | 1481 | 244 | 17 | 64 | 1256 | 0 | 652 | 755 | 680 | 1 |
| **Leicester** | 981 | 582 | 282 | 487 | 1600 | 0 | 499 | 757 | 761 | 0 |
| **Liverpool** | 1087 | 810 | 18 | 136 | 1311 | 1 | 1235 | 831 | 564 | 1 |
| **Manchester** | 780 | 866 | 2 | 349 | 1237 | 1 | 883 | 829 | 587 | 1 |
| **Mansfield** | 232 | 1265 | 4 | 709 | 923 | 0 | 310 | 553 | 568 | 0 |
| **Middlesbrough** | 73 | 1419 | 185 | 105 | 1099 | 0 | 241 | 305 | 416 | 0 |
| **Newcastle** | 871 | 925 | 367 | 34 | 1281 | 0 | 480 | 566 | 573 | 2 |
| **Norwich** | 1319 | 373 | 197 | 432 | 1024 | 0 | 328 | 348 | 590 | 0 |
| **Nottingham** | 1288 | 217 | 14 | 437 | 1099 | 2 | 695 | 721 | 776 | 0 |
| **Oxford** | 1444 | 190 | 7 | 691 | 1525 | 2 | 1083 | 1071 | 842 | 0 |
| **Preston** | 9 | 1516 | 3 | 7 | 485 | 0 | 344 | 494 | 565 | 1 |
| **Reading** | 339 | 1401 | 107 | 84 | 1153 | 0 | 401 | 165 | 717 | 0 |
| **Sheffield** | 1317 | 595 | 3 | 671 | 1132 | 1 | 478 | 609 | 523 | 0 |
| **Slough** | 1278 | 135 | 35 | 9 | 1628 | 0 | 1140 | 768 | 702 | 0 |
| **Stoke** | 0 | 1543 | 3 | 1077 | 1359 | 0 | 613 | 523 | 444 | 0 |
| **Swindon** | 60 | 1324 | 197 | 88 | 1519 | 0 | 408 | 685 | 467 | 2 |
| **York** | 1181 | 672 | 392 | 553 | 1199 | 4 | 459 | 595 | 554 | 0 |
| **Edinburgh** | 1128 | 564 | 564 | 584 | 1382 | 0 | 1283 | 817 | 886 | 0 |
| **Glasgow** | 941 | 584 | 377 | 692 | 1180 | 0 | 812 | 723 | 549 | 0 |
| **Cardiff** | 1422 | 411 | 50 | 1200 | 1595 | 1 | 739 | 462 | 726 | 536 |
| **Swansea** | 927 | 598 | 175 | 1085 | 682 | 1 | 157 | 88 | 256 | 0 |
| **Wrexham** | 0 | 1446 | 97 | 980 | 2 | 2 | 224 | 180 | 347 | 1 |

### Sample size

To determine the adequate sample size for the second and the third stage, we used Cambridge as a test case. We sampled 2000 random locations in Cambridge using the GIS network. For each location, we accessed historical database of panoramas using the ‘streeview’ package. Out of 2000 locations, 114 had no panorama. Among the rest of 1886 location (2000 minus 114), the yearly distribution of panoramas is 61% (2008), 38% (2010), 16% (2011), 76% (2012), 85% (2014), 53% (2015), and 46% (2016). The percentages do not total to 100% as many locations have panoramas for more than a year—on an average, 3.8 unique years and varying from 1 to 7.

For the years 2010–2012, we selected 1572 unique locations as the rest (1886 minus 1572) did not have any image corresponding to the 3-year period. For the locations with panoramas of more than one of the three years, the selection was based on the preference order of 2011>2012>2010. For each selected location, we accessed four images corresponding to the headings of 0, 90, 180 and 270 degrees, thus covering the 360-degree view. The rest of the parameters in the API were as described earlier.

Next, we observed the images and collected the data using the web-based questionnaire. The locations were randomised so that they are not in any particular order and therefore any set of images are randomly scattered throughout the city. We expressed the outputs of the questionnaire as proportions, calculated by dividing the observed number of different sub categories (such as pedestrian: 1-3; pedestrian: 4-6) by the total number of images observed. After completing every set of 100 images, we plotted the proportions of cumulative number of observed road user sub-categories along with the cumulative number of total images observed (100, 200, 300, and so on). We found that the proportions of all the sub categories stabilised around 1000 images.

## Inter-Rater Agreement

### Methods

The four Research Assistants (RAs) assessed 500 random GSV images from Birkenhead city, apart from those used in the comparisons against the Census and APS estimates. We evaluated the inter-rater agreement based on the reported presence or absence of pedestrians, cyclists, parked cycles, cars, buses, motorcycles, and van/trucks.

We computed the percentage of images in which all RAs achieved the same answer (percentage agreement), intraclass correlation coefficient (ICC), and Finn’s coefficient. For the latter two, we used a two-way model to consider images and RAs as random effects. For ICC, unit of analysis was the average of the 500 ratings. ICCs ranging from 0.60 to 0.74 may be interpreted as good agreement, whereas values above this range are deemed as excellent (see Tables B and C).

Analyses were conducted in R version 3.4.1, using the ‘irr’ package, version 0.84.

### Results

| **Table B. Number of images containing road users reported by Research Assistant (RA) (500 images).** | | | | | | | | | | | |
| --- | --- | --- | --- | --- | --- | --- | --- | --- | --- | --- | --- |
| **Road users** | **RA 1** | |  | **RA 2** | |  | **RA 3** | |  | **RA 4** | |
|  | **n** | **%** |  | **n** | **%** |  | **n** | **%** |  | **n** | **%** |
| Pedestrians | 73 | 14.6 |  | 67 | 13.4 |  | 79 | 15.8 |  | 68 | 13.6 |
| Cyclists | 3 | 0.6 |  | 1 | 0.2 |  | 2 | 0.4 |  | 2 | 0.4 |
| Parked cycles | 3 | 0.6 |  | 1 | 0.2 |  | 1 | 0.2 |  | 2 | 0.4 |
| Cars | 381 | 76.2 |  | 382 | 76.4 |  | 388 | 77.6 |  | 381 | 76.2 |
| Buses | 3 | 0.6 |  | 3 | 0.6 |  | 3 | 0.6 |  | 4 | 0.8 |
| Motorcycles | 5 | 1.0 |  | 5 | 1.0 |  | 7 | 1.4 |  | 8 | 1.6 |
| Vans/trucks | 78 | 15.6 |  | 82 | 16.4 |  | 69 | 13.8 |  | 87 | 17.4 |

| **Table C. Inter-rater agreement between the four Research Assistants (500 images)** | | | | | | | |
| --- | --- | --- | --- | --- | --- | --- | --- |
| **Road users** | **Percentage**  **agreement** |  | **ICC** | |  | **Finn’s coeff.** | |
|  |  |  | **Coeff.** | **95%CI** |  | **Coeff.** | **p-value** |
| Pedestrians | 73.2 |  | 0.75 | 0.72 – 0.79 |  | 0.72 | <1^-10^ |
| Cyclists | 99.0 |  | 0.66 | 0.61 – 0.71 |  | 0.99 |  |
| Parked cycles | 99.0 |  | 0.61 | 0.55 – 0.67 |  | 0.99 |  |
| Cars | 60.6 |  | 0.76 | 0.72 – 0.79 |  | 0.60 |  |
| Buses | 98.2 |  | 0.64 | 0.58 – 0.69 |  | 0.98 |  |
| Motorcycles | 97.2 |  | 0.74 | 0.70 – 0.78 |  | 0.97 |  |
| Vans/trucks | 69.2 |  | 0.73 | 0.69 – 0.76 |  | 0.68 |  |
| ICC: Intraclass correlation coefficient. Coeff.: Coefficient. 95%CI: 95% confidence interval. | | | | | | | |

## Questions in Active People Survey

*Walking*

1. On how many days in the last four weeks have you done at least one continuous walk lasting at least 10 minutes?
2. On the days that you walked, what was the total length of time you USUALLY spent walking during the course of the day? (Please only include walks of at least 10 minutes).
3. On how many of those days did you walk for the purpose of health or recreation not to get from place to place again please exclude time spent walking around shops?
4. On these days, what was the total length of time you USUALLY spent walking for the purpose of health or recreation, not to get from place to place, during the course of the day? (Please only include walks of at least 10 minutes).

*Cycling*

1. On how many days in the last 4 weeks have you done any cycling?
2. On the days that you cycled, what was the total length of time you USUALLY spent cycling during the course of the day?
3. On how many of those days did you cycle for the purpose of health, recreation, training or competition not to get from place to place?
4. Thinking only about continuous cycle rides for the purpose of health, recreation, training or competition not to get from place to place, how long do you usually cycle for?

**Total walking and cycling:** Prevalence of people who have done at least one continuous walk lasting at least 10 minutes in the previous four weeks. Prevalence of people who have done any cycling in the previous four weeks.

People reported days they have done at least one continuous walk lasting at least 10 minutes and any cycling in the previous four weeks. These values were divided by four to get the average number of days per week people walked and cycled.

People reported the total time they spent walking and cycling on a usual day. These values were multiplied by the average days per week to get the average weekly volume of walking and cycling.

**Utility (transport-related) walking and cycling:** People reported how many days they walked and cycled for recreational or health purposes in the previous four weeks. These values were subtracted from the reported walking and cycling days for any purpose to get the days people walked and cycled exclusively for transportation purposes. The same subtraction step has been done to get walking and cycling daily duration for transportation purposes.

Prevalence, average days per week, and average volume per week of transport-related walking and cycling were obtained the same way as for total walking and cycling.

## Seasonality of GSV images


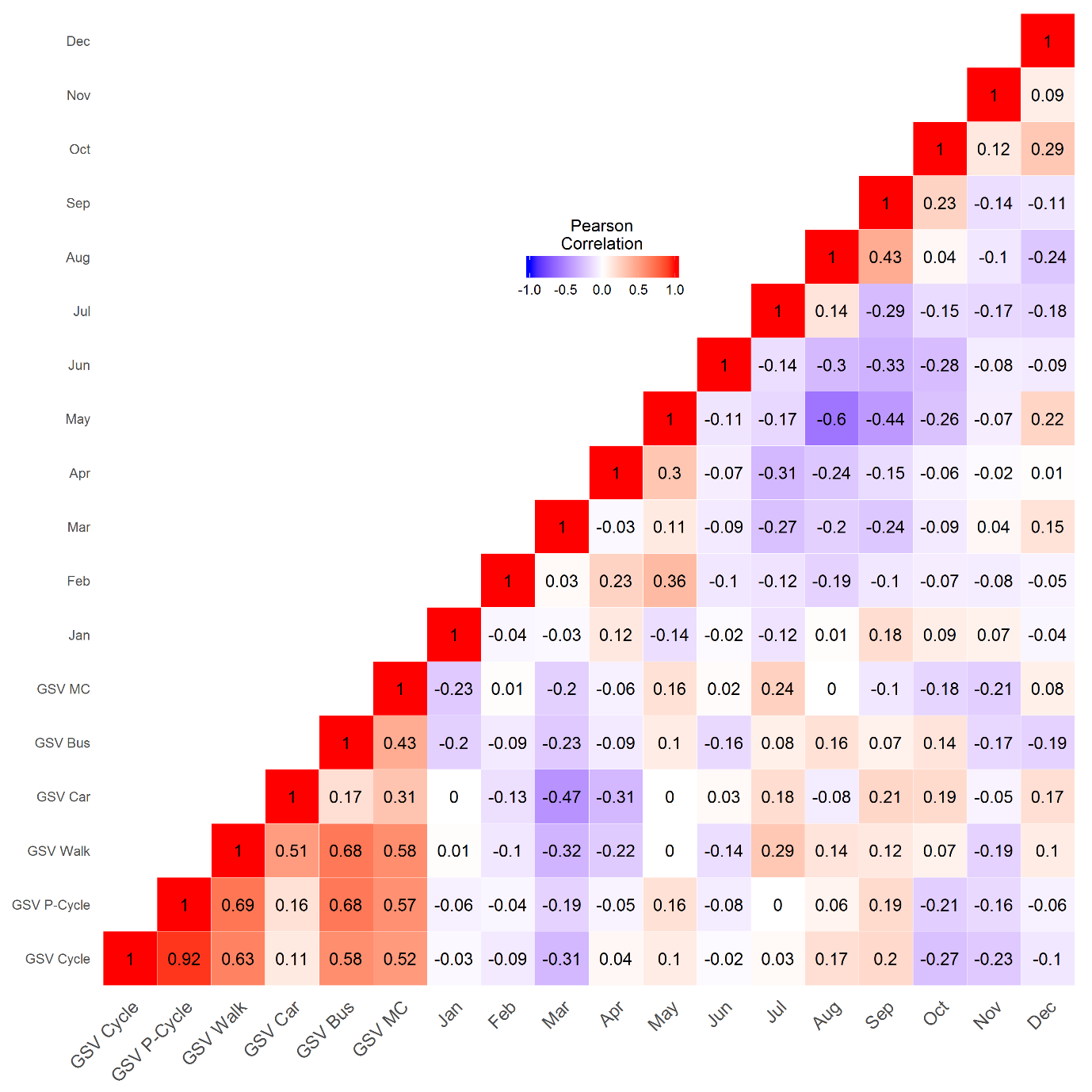


**Fig B. Correlation plot between GSV measures and monthly proportions of images**

## R script for calculating standardised error in beta regression model

datapoints <- dim(data1)[1]

loocv_residuals <- c()

for(i in 1:datapoints){

weights <- rep(1,datapoints)

weights[i] <- 0

gy <- betareg(y~x, data = data,weights=weights)

gy$weights <- rep(1,datapoints)

loocv_residuals[i] <- residuals(gy, type = "sweighted2")[i]

}

median(abs(loocv_residuals))

mean(abs(loocv_residuals))

## Regression models

**Table D. Linear Regression models**

|  | **Model 11*** | **Model 12** | **Model 13*** | **Model 15*** |
| --- | --- | --- | --- | --- |
|  | **APS Days All Cycl** | **APS Days All Walk** | **APS Dur Utly Cycl** | **APS Dur All Cycl** |
| **(Intercept)** | 0.007 | 3.302 | 0.033 | 0.207 |
| **GSV Walk** |  | 0.001 |  |  |
| **GSV Cycle** | 0.018 |  | 0.008 | 0.008 |
| **MAE** | 0.13 | 0.14 | 0.07 | 0.10 |
| **MDAE** | 0.07 | 0.1 | 0.03 | 0.06 |

* robust linear regression models

1. https://www.google.co.uk/maps/@51.5008694,-0.1039006,3a,75y,54.5h,90t/data=!3m7!1e1!3m5!1s**uydT7NJJHBREgcesdeitrQ**!2e0!6s%2F%2Fgeo1.ggpht.com%2Fcbk%3Fpanoid%3D**uydT7NJJHBREgcesdeitrQ**%26output%3Dthumbnail%26cb_client%3Dmaps_sv.tactile.gps%26thumb%3D2%26w%3D203%26h%3D100%26yaw%3D41.11041%26pitch%3D0%26thumbfov%3D100!7i13312!8i6656?hl=en [↑](#footnote-ref-1)
